# Clinicopathological and prognostic value of SNHG6 in cancers: a systematic review and a meta-analysis

**DOI:** 10.1186/s12885-020-06850-0

**Published:** 2020-04-22

**Authors:** Shuo Zhang, Dandan Qiu, Xiaohong Xie, Yong Shen

**Affiliations:** 1 Department of Breast Surgery, The First Affiliated Hospital of Zhejiang Chinese Medical University (Zhejiang Provincial Hospital of Traditional Chinese Medicine), Hangzhou, 310006 China; 2 Department of Urology, The First Affiliated Hospital of Zhejiang Chinese Medical University (Zhejiang Provincial Hospital of Traditional Chinese Medicine), Hangzhou, 310006 China

**Keywords:** LncRNA SNHG6, Cancer, Clinical outcome, Meta-analysis

## Abstract

**Background:**

Dysregulation of the long non-coding RNA small nucleolar RNA host gene 16 (lncRNA SNHG6) has been found in multiple cancers. However, a definite conclusion on the clinical value of lncRNA SNHG6 expression in human cancers has not been determined. The purpose of the present meta-analysis was to comprehensively elucidate the association between SNHG6 expression and clinical outcomes in cancers.

**Methods:**

A systematic search was performed through the PubMed, Web of Science, Chinese National Knowledge Infrastructure (CNKI), and Wangfang databases for relevant studies. The pooled hazard ratios (HRs) with 95% confidence intervals (CIs) were collected to estimate the prognostic value, and the odds ratios (ORs) with 95% CIs were used to evaluate the relationship between lncRNA SNHG6 expression and clinicopathological features, including tumor invasion depth, lymph node metastasis (LNM), distance metastasis (DM), and TNM stage.

**Results:**

In total, 914 patients from 13 studies were included in this meta-analysis. The pooled results suggested that evaluated SNHG6 expression could predict an unfavorable overall survival (OS) (HR = 2.04, 95% CI:1.56–2.52) with no heterogeneity (I^2^ = 0.0%, *p* = 0.996). Subgroup analysis indicated a significant association between high SNHG6 expression and shorter OS in those studies with digestive system cancers (HR = 2.05, 95% CI: 1.47–2.62), or with sample size < 70 (HR = 2.70, 95% CI: 1.29–4.11), or with multivariate analysis (HR = 2.04, 95% CI: 1.44–2.64). Moreover, elevated SNHG6 expression was positively associated with tumor invasion depth (OR = 1.76, 95% CI: 1.18–2.63), LNM (OR = 1.60, 95% CI: 1.18–2.17), DM (OR = 1.90, 95% CI: 1.37–2.64) and advanced TNM stage (OR = 1.88, 95% CI: 1.36–2.60) in patients with cancers.

**Conclusions:**

High lncRNA SNHG6 expression was correlated with tumor invasion depth, LNM, DM, and advanced TNM stage, suggesting that SNHG6 may serve as a promising prognostic biomarker of human cancers.

## Background

Cancer is one of the major public health issues and one of the leading causes of morbidity and mortality worldwide. In 2018, there were a predicted 18.1 million new cases and 9.6 million deaths of cancers worldwide based on a report by the International Agency for Research on Cancer [1]. Although significant advances in the diagnosis and treatment of tumors over the past decade, the 5-year survival rate remains worse in most patients with cancer, mainly due to the lack of ideal biomarkers for the early detection and effective treatment of tumors. Therefore, it is urgent to develop promising forecasting biomarkers in precise therapy and prognostication of cancer.

Long non-coding RNAs (lncRNAs) is an important member of non-coding RNAs (ncRNAs) comprising a transcription length of more than 200 nucleotides but not coding proteins [2, 3]. Numerous studies have suggested that lncRNAs play vital roles in various physiological and pathological process of cancers, including cell proliferation, migration, invasion, and metabolism by functioning as oncogene or tumor suppressor [4–6]. Furthermore, growing evidence has demonstrated that lncRNAs can be recognized as tumor-specific prognostic predictors for some cancers, and recent meta-analyses have suggested several lncRNAs correlated with prognosis and chinicopathological features as candidates for precise prognosis prediction of cancers, such as DANCR [7], CRNDE [8] and MVIH [9].

LncRNA small nucleolar RNA host gene 16 (SNHG6), also known as U87HG, locates in human chromosome 8q13.1. Previous studies have demonstrated that SNHG6 is overexpressed in different kinds of cancers, such as renal cell carcinoma [10], gastric cancer [11], breast cancer [12], and colorectal cancer [13]. It has been shown to promote proliferation, migration, invasion, and/or epithelial-mesenchymal transition (EMT) in multiple types of cancerous cells [11]. Moreover, evaluated SNHG6 expression has been found to be associated with clinicopathologic characteristics [10, 14, 15]. Consequently, cancer patients with high lncRNA SNHG6 expression tend to have a poor prognosis. However, given the discrete outcomes and limited sample size in current studies, we performed this meta-analysis to evaluate the potential value of SNHG6 as a promising prognostic biomarker in human cancers.

## Methods

### Literature searching strategies

To retrieve potentially eligible studies on the clinical value of SNHG6 expression in human cancers, the comprehensive literature search was performed in the PubMed, Web of Science, and two Chinese literature database: WangFang and CNKI from inception to August 19, 2019. The following keywords were used in combination for search: (“cancer” OR “tumor” OR “neoplasm” OR “carcinoma”), (“prognosis” OR “diagnosis” OR “survival”) and (“SNHG6” OR “small nucleolar RNA host gene 6”). The reference lists of the relevant studies were screened manually for potentially missing literature.

### Inclusion and exclusion criteria

The assessment of eligible articles was performed by two independent researchers (Qiu and Zhang) according to the inclusion and exclusion criteria, and discrepancies between them were resolved via negotiation. Inclusion criteria were as follows: 1) studies reporting the relationship between lncRNA SNHG6 expression and clinicopathological characteristics and prognosis, 2) human cancer, 3) patients were grouped based on the level of SNHG6 expression, 4) studies providing available data for extracting or calculating HRs and 95%CIs for OS. Exclusion criteria were as follows: 1) reviews, letters, conference reports, and animal studies; 2) studies without available survival data.

### Data extraction and quality assessment

Two researchers independently examined all eligible studies and extracted carefully the essential information, including author, year of publication, country, type of cancer, sample size, the method for detecting SNHG6 expression, outcomes, HRs and 95% CIs, as well as clinicopathologic characteristics. The enrolled literatures were then qualified by PRISMA checklists (Additional file 1: Table S1). HRs and 95%CIs analyzed by multivariable analysis had priority to be chosen when available. For those studies only containing the Kaplan-Meier curve, Engauge Digitizer Version 10.8 (http://markummitchell.github.io/engauge-digitizer/) and published method were performed to calculate survival data and obtain HRs and 95%CIs indirectly [16]. The Newcastle-Ottawa Scale (NOS) was used to evaluate the quality of the included studies, while score ≥ 6 represents high quality.

### Statistical analysis

All extracted data were analyzed using STATA software version 15.0 (StataCorp LLC, College Station, TX, USA). The association between SNHG6 expression and prognosis in cancers were evaluated by the pooled HRs and corresponding 95% CIs. Pooled ORs and corresponding 95%CIs were used to assess the correlation of lncRNA SNHG6 with clinicopathological characteristics. The heterogeneity was analyzed using the Chi-squared test and I^2^ statistics. The fixed-effect model was applied when I^2^ > 50% and *P* > 0.05. Otherwise, the random-effect model was used [7]. The publication bias was assessed by the Funnel plots and Begg’s test, and sensitivity analysis was performed to examine the robustness of results. *P*-value < 0.05 were recognized as statistical significance.

## Results

### Study selection and characteristics

**Fig. 1:**
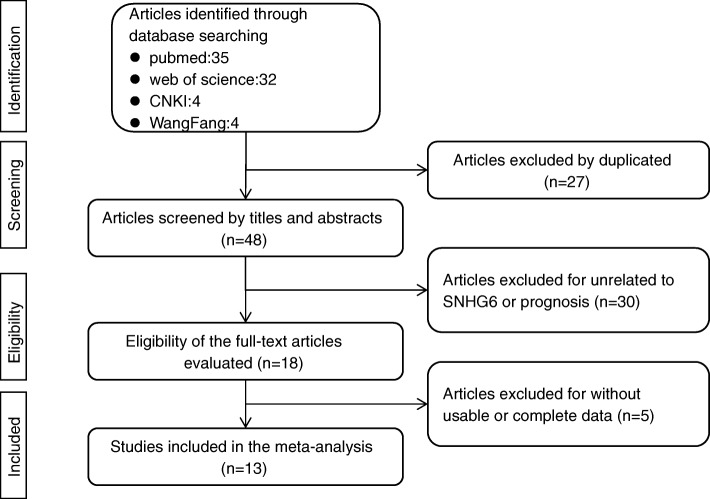
Flow chart of the study selection procedure in this meta-analysis

The process of literature search and selection was detailed in Fig. 1. In total, 75 potentially relevant records were obtained. After excluding the duplicated and unqualified papers, 13 studies involving 914 patients with 8 different types of cancers were enrolled in this meta-analysis ultimately [5, 10, 13, 14, 17–25]. These included studies comprised renal cell carcinoma [10], glioma [14, 18], hepatocellular carcinoma [17], colorectal cancer [13, 20, 21, 24], ovarian clear cell carcinoma [19], gastric cancer [11], esophageal squamous cell carcinoma [11] and osteosarcoma [23, 25].

The major characteristics of the eligible articles were summarized in Table 1. All included studies were conducted in China and published from 2017 to 2019. The sample size of the included studies ranged from 30 to 141. The expression level of lncRNA SNHG6 was detected by quantitative real-time polymerase chain reaction (qRT-PCR) in all studies and all patients of each study were divided into high and low groups based on the expression of SNHG6. Of the 13 studies, 6 studies recorded the HR and corresponding 95% CI for OS, and data on OS of the other 7 studies were extrapolated through Kaplan-Meier curves indirectly. Additionally, all included studies were considered high quality because of the NOS scores were more than 6 for each study.

**Table 1:** Characteristics of studies included in the meta-analysis

Author	Year	Country	Tumor Type	Sample Size	Cat-off value	Follow-up (month)	SNHG6 expression				Detection Method	Outcome measures	Survival Analysis	HR estimated method	NOS
							High		Low						
							LNM	DM	LNM	DM					
An HX	2018	China	RCC	81	FC > 1	80(total)	32	NA	11	NA	qRT-PCR	OS	Univariate; multivariate	Directly	8
Cai G	2018	China	Glioma	58	median	60(total)	NA	NA	NA	NA	qRT-PCR	OS	Univariate	Indirectly	8
Chang L	2016	China	HCC	80	median	36(total)	NA	NA	NA	NA	qRT-PCR	OS/RFS	Univariate; multivariate	Directly	7
Li M	2017	China	CRC	74	median	58(median)	NA	NA	NA	NA	qRT-PCR	OS	Univariate; multivariate	Directly	6
Meng Q	2018	China	Glioma	71	median	60(total)	NA	NA	NA	NA	qRT-PCR	OS	Univariate	Indirectly	7
Wu Y	2018	China	OCCC	48	median	70(total)	10	10	8	17	qRT-PCR	OS/PFS	Univariate	Indirectly	7
Xu M	2019	China	CRC	120	median	90(total)	18	41	7	55	qRT-PCR	OS/DFS	Univariate; multivariate	Directly	8
Yan K	2017	China	GC	78	median	60(total)	21	13	19	21	qRT-PCR	OS	Univariate	Indirectly	8
Yu C	2019	China	CRC	141	median	60(total)	12	62	5	69	qRT-PCR	OS/RFS	Univariate; multivariate	Directly	6
Zhang YL	2019	China	ESCC	75	median	60(total)	31	31	13	31	qRT-PCR	OS	Univariate	Indirectly	8
Zheng LL	2018	China	osteosarcoma	58	mean	60(total)	NA	8	NA	23	qRT-PCR	OS	Univariate; multivariate	Directly	8
Zhu X	2019	China	osteosarcoma	30	median	60(total)	11	12	6	12	qRT-PCR	OS	Univariate	Indirectly	7
Zhu YK	2018	China	CRC	40	median	60(total)	16	13	5	19	qRT-PCR	OS	Univariate	Indirectly	8

Notes: *RCC* renal cell carcinoma, *HCC* hepatocellular carcinoma, *CRC* colorectal cancer, *OCCC* ovarian clear cell carcinoma, *GC* gastric cancer, *ESCC* esophageal squamous cell carcinoma, *qRT-PCR* quantitative real-time PCR, *OS* overall survival, *RFS* relapse-free survival, *PFS* progression-free survival, *DFS* disease-free survival, *NOS* The Newcastle-Ottawa Scale, *FC* Fold-change

### Prognostic value of SNHG6 expression in solid cancers

**Fig. 2:**
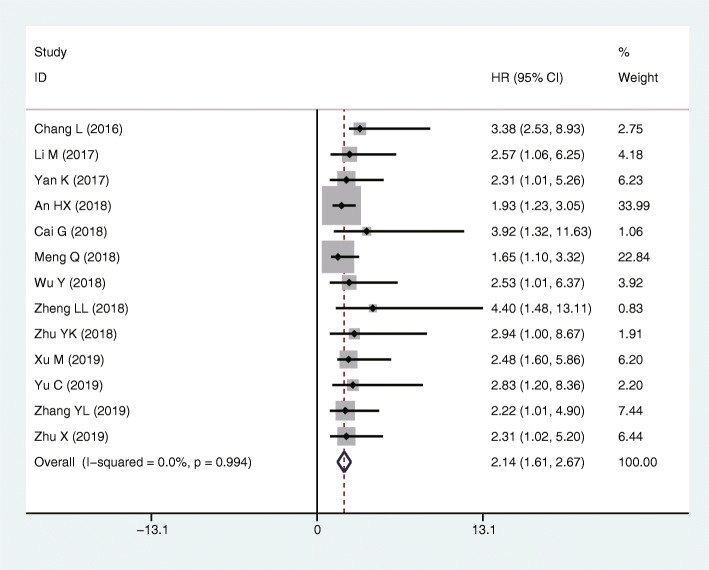
Forest plot of the HRs for the correlation between high lncRNA SNHG6 expression and OS

**Fig. 3:**
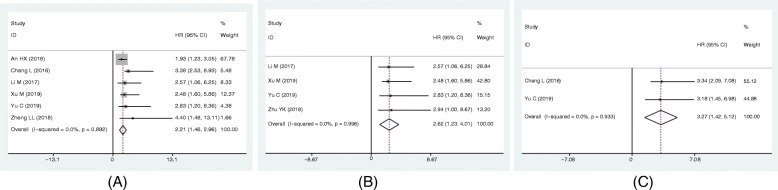
Forest plots of the HRs for the association between SNHG6 expression and (**a**) independent predictive factor for OS, (**b**) OS of patients with colorectal cancer, and (**c**) RFS of cancer patients

The HR and 95%CI from 13 studies (including 914 patients) was combined to determine the association between lncRNA SNHG6 expression and OS. As shown in Fig. 2, no obvious heterogeneity was observed among the studies (*I*^2^ = 0.00%, *p* = 0.994). Therefore, a fixed-effect model was applied. The pooled HR was 2.14 (95% CI: 1.61 ~ 2.67, *p* < 0.001), indicating that patients with increased expression of lncRNA SNHG6 predicted a poor OS in 8 types of human cancers (Fig. 2). Meanwhile, the independent prognostic value of SNHG6 expression was also assessed based on the multivariate analysis in 6 studies with 514 patients (Fig. 3a). The pooled results revealed that SNHG6 expression was an independent prognostic factor for OS in cancer patients (HR = 2.21, 95% CI: 1.46–2.96, *p* < 0.001; *I*^2^ = 0.0%, *p* = 0.892). Particularly, for colorectal cancer, the pooled HR was 2.62 with 95% CI (1.23–4.01) (Fig. 3b). In addition, the prognostic value of SNHG6 expression for RFS was also assessed in 2 studies with 221 patients (Fig. 3c). The pooled result indicated that increased SNHG6 expression was associated with a poor RFS in hepatocellular carcinoma and colorectal cancer (HR = 3.27, 95% CI: 1.42–5.12, *p* < 0.001; *I*^2^ = 0.0%, *p* = 0.93).

**Fig. 4:**
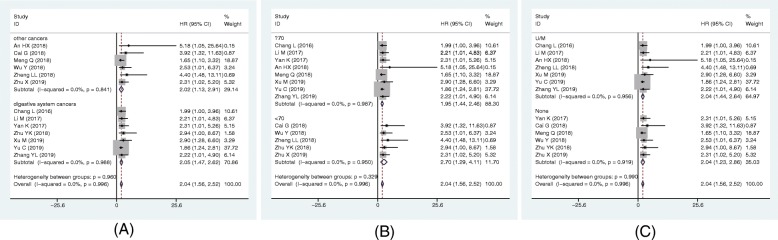
Forest plots of the subgroup analysis evaluating HRs of lncRNA SNHG6 for OS by the factors of (**a**) cancer type, (**b**) sample size, and (**c**) HR estimation method

Furthermore, subgroup analysis of OS was also performed according to types of tumor, sample size, and survival analysis, as shown in Fig. 4. Stratified analysis showed that SNHG6 overexpression could predict unfavourable OS in digestive system (HR = 2.5, 95% CI: 1.57–3.48, *p* < 0.001; *I*^2^ = 0.0%, *p* = 0.998), and other system (HR = 1.97, 95% CI: 1.33–2.61, *p* < 0.001; *I*^2^ = 0.0%, *p* < 0.874). And we also found that evaluated SNHG6 level significantly related to unfavorable OS in the studies with sample size < 70 (HR = 2.70, 95%CI: 1.29–4.11, *p* < 0.001; I^2^ = 0.0%, *p* = 0.950), as well as those with sample size ≥70 (HR = 2.05, 95% CI: 1.48–2.62, *p* < 0.001; *I*^2^ = 0.0%, *p* = 0.970). Moreover, higher SNHG6 expression could predict poorer outcome in the studies carried out by univariate and multivariate (U/M) analysis (HR = 2.21, 95% CI: 1.46–2.96, *p* < 0.001; *I*^2^ = 0.0%, *p* = 0.892), as well as those without U/M analysis (HR = 2.07, 95% CI: 1.32–2.82, *p* < 0.001; *I*^2^ = 0.0%, *p* = 0.961).

### Correlation between SNHG6 and clinicopathologic characteristics

**Fig. 5:**
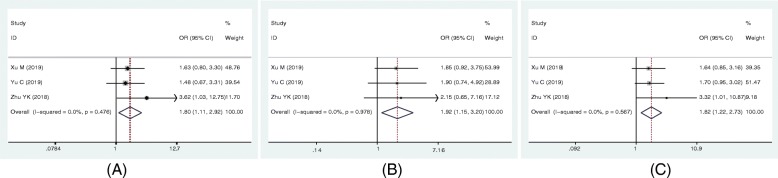
Forest plots of the included studies evaluating the correlation between SNHG6 expression and clinicopathological characteristics of patients with colorectal cancer. **a** LNM; (**b**) DM, and (**c**) TNM

A correlation between lncRNA SNHG6 expression and clinicopathological features were obtained from OR analysis. The combined results were shown in Table 2. The pooled results from 4 studies indicated that the high lncRNA SNHG6 expression was related to tumor invasion depth (OR = 1.76, 95% CI: 1.18–2.63, *p* = 0.006, *I*^2^ = 0.24%), lymph node metastasis (LNM) (OR = 1.60, 95% CI: 1.18–2.17, *p* = 0.002, *I*^2^ = 5.57%), distant metastasis (DM) (OR = 1.90, 95% CI: 1.37–2.64, *p* < 0.001, *I*^2^ = 0.73%) and advanced TNM stage (OR = 1.88, 95% CI: 1.36–2.60, *p* < 0.001, *I*^2^ = 1.3%). In addition, for colorectal cancer, the pooled results also suggested that the elevated SNHG6 expression was associated with LNM (OR = 1.80, 95% CI: 1.11–2.92; Fig. 5a), DM (OR = 1.92, 95% CI: 1.15–3.20; Fig. 5b), and TNM (OR = 1.82, 95% CI: 1.22–2.73; Fig. 5c). Therefore, our meta-analysis suggested that lncRNA SNHG6 overexpression was associated with advanced clinicopathological characteristics.

**Table 2:** Meta-analysis of association between evaluated SNHG6 expression and four clinicopathological characteristics

Clinicopathological parameters	Studies (n)	Patients (n)	OR (95% CI)	*p*-value	Heterogeneity		
					*I*^*2*^	P_h_	Model
Tumor invasion depth (T_3–4_ VS T_1–2_)	4	309	1.76 (1.18–2.63)	0.006	0.24	0.972	fixed
Lymph node metastasis (Yes vs No)	8	610	1.60 (1.18–2.17)	0.002	5.57	0.591	fixed
Distant metastasis (Yes vs No)	8	590	1.90 (1.37–2.64)	< 0.001	0.73	0.998	fixed
TNM stage (III-IV vs I-II)	6	484	1.88 (1.36–2.60)	< 0.001	1.3	0.935	fixed

### Publication bias and sensitivity analysis

**Fig. 6:**
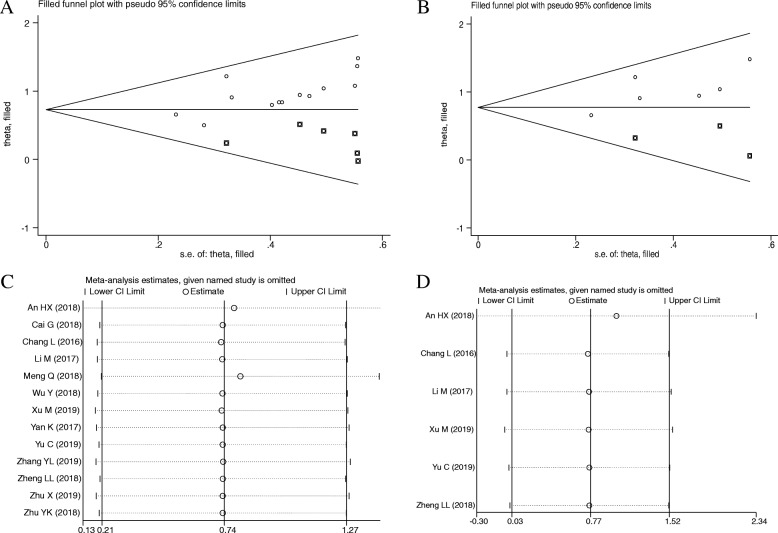
Publication bias and sensitivity analysis for OS in this meta-analysis. **a** Begg’s funnel plot analysis for potential publication bias among included eligible studies; **b** Begg’s funnel plots of the included studies for independent predictive factor for OS; **c** Sensitivity analysis of the included studies for OS; **d** Sensitivity analysis of the included studies for independent predictive factor for OS

The publication bias was evaluated by Begg’s funnel plot and Egger’s linear regression tests in the present meta-analysis. Visual inspection of the funnel plot revealed the absence of asymmetry (Fig. 6a), as well as Egger’s test showed probable evidence for publication bias in our meta-analysis (*t* = 7.12, *p* < 0.001). Therefore, we preformed trim and fill analysis with a fixed-effect model to assessed the impact of potential publication bias. The pooled analysis incorporation the hypothetical studies continued to show a significant association between SNHG6 expression with OS in human cancers (corrected HR = 2.07, 95% CI: 1.73–2.48, *p* < 0.001). As shown in Fig. 6b, We also performed trim and fill analysis when evaluating the independent prognostic value of SNHG6 expression for OS in cancers because of the present of asymmetry of funnel plot and the result of Egger’s test (*t* = 8.52, *p* = 0.001). The pooled data also showed a relationship between SNHG6 overexpression with poor OS in human cancers (corrected HR = 2.16, 95% CI: 1.70–2.75, *p* < 0.001). Publication bias in the RFS groups was not analyzed due to the small number of studies.

The sensitivity analysis was carried out by removing each study in turn from the pooled analysis to examine the impact of the removed study on the overall HRs. As shown in Fig. 6C-D, the pooled HR was not significantly changed when removing any of the included studies, suggesting the robustness of the results in the present research.

## Discussion

Long non-coding RNAs comprise a vast less explored region of the human genome, which may play crucial roles in carcinogenesis and cancer development. Recently, more evidence has emerged that aberrant expression of lncRNAs present in a variety of human cancers and has promoted the development of lncRNAs-based diagnosis and therapies. Accumulating studies have reported the up-regulation of lncRNA SNHG6 in many cancers, such as breast cancer [15], hepatocellular carcinoma [26], and gastric cancer [11]. Currently, lncRNA SNHG6 have been confirmed as a dysregulated oncogene in human tumors. Its overexpression is associated with LNM, DM, advanced TNM stage, and poor prognosis in patients with cancers. Moreover, silencing of lncRNA SNHG6 significantly suppressed proliferation, migration, metastasis, and invasion of cancerous cells [15, 19, 25, 26]. Due to its oncogenic potential, lncRNA SNHG6 is defined as a carcinogenic lncRNA in many cancers. Furthermore, lncRNA SNHG6 has gained attention recently as a potential biomarker for predicting cancer prognosis. Here we conducted this meta-analysis to evaluate the prognostic value of lncRNA SNHG6 and its association with clinicopathological parameters in human cancers.

A total of 13 eligible studies with 914 patients meeting inclusion criteria were included in this meta-analysis. Our results demonstrated that lncRNA SNHG6 overexpression was significantly associated with poor outcome and could serve as an unfavorable prognostic biomarker in cancer patients. Furthermore, we evaluated the relationship between evaluated SNHG6 with four clinicopathological characteristics, including tumor invasion depth, LNM, DM, and TNM stage. The pooled data revealed that increased expression of SNHG6 was significantly associated with tumor invasion depth, LNM, DM, and advanced TNM stage, indicating that evaluated SNHG6 expression correlated with advanced clinicopathological characteristics. To sum up, our observations provided convincing evidence to support SNHG6 as a favorable prognostic biomarker for human cancers.

Up till now, the underlying molecular mechanisms involved in SNHG6 interactions in cancers are complex and remain poorly understood. Recent studies have demonstrated that SNHG6 could provide specific functional scaffolds for regulatory complexed, such as enhancer of zeste 2 polycomb repressive complex 2 sub-unit (EZH2). It was approved that SNHG6 played an oncogenic role in gastric cancer through silencing expression at a transcriptional level by recruiting enhancer of EZH2 to the promoter of p27 [11]. Moreover, in colorectal cancer, SNHG6 functioned as an oncogene to interact with UPF1 to activate TGF-β/Smad signaling pathway, promotes proliferation, invasion, and migration [27], and our results also suggested that evaluated SNHG6 significantly related to unfavorable prognosis and advanced clinicopathological characteristics for patients with colorectal cancer.

Additionally, an increased number of studies have demonstrated that SNHG6 could serve as a competing endogenous RNA (ceRNA) to inhibit functions of miRNAs. For example, in breast cancer, Li et al. have found that up-regulation of SNHG6 contribute to cancer progression by SNHG6/miR-26a/VASP axis [15]. In glioma, Meng et al. have demonstrated that SNHG6 function as a ceRNA for miR-101-3p to induce tumor growth and progression [18]. Recent discoveries have revealed that dysregulated SNHG6 can lead to aberrant genome-wide hypomethylation by inhibiting SAMe production [28]. Furthermore, SNHG6 regulated ZEB1 expression by competitively binding miR-101-3p in hepatocellular carcinoma [11]. Collectively, it has been also revealed that SNHG6 functioned as ceRNA by competitively binding miR-139-5p [26], miR-15a [29], miR-4465 [19], miR-181a-5p [21], miR-214 [21], miR-26a-5p [23], miR-760 [24], miR-125b [24], and miR-1297 [30]. Therefore, further studies are required to fully appreciate the functions of SNHG6 in the progression of cancers.

However, there were several limitations in our meta-analysis. Firstly, owing to the small sample size of the included studies, we failed to pool results by one single type of cancer. Therefore, we assessed the prognostic value of SNHG6 expression based on the digestive system and non-digestive system. Secondly, all included studies were carried out in China, which would generate a region bias. Thus, further large-scale and well-designed research were required to confirm the clinical value of SNHG6 in different ethnicities. Thirdly, most of the HRs and 95% CIs were calculated indirectly based on the survival curve, which might result in the overestimation or underestimation of the clinical significance of SNHG6 expression in many cancers. Moreover, the follow-up period of cancer patients, as well as the cutoff value, are inconsistent between different literature reports, which will also have certain impacts on the analysis results.

## Conclusion

In conclusion, this meta-analysis demonstrated that SNHG6 overexpression is correlated with shorter overall survival, as well as tumor invasion depth, lymph node metastasis, distant metastasis, and advanced TNM stage. Therefore, SNHG6 may potentially be used as a novel prognostic biomarker in human cancers. In the future, more well-designed studies with larger sample size are needed to validate the prognostic value of SNHG6 in different cancers of various ethnic populations.

## Supplementary information

**Additional file 1.** PRISMA checklist.

## Data Availability

All data analyzed during this study are included in this published article.

## Abbreviations

LncRNAs: Long non-coding RNAs
HRs: hazard ratios
95% CI: 95% confidence interval
OR: Odds ratios
LNM: Lymph node metastasis
DM: Distance metastasis
ceRNA: Competing endogenous RNA
EMT: Epithelial-mesenchymal transition
OS: Overall survival
RFS: Relapse free survival
SNHG6: Small nucleolar RNA host gene 6
NOS: The Newcastle-Ottawa Scale
